# The Role of Sex in Memory Function: Considerations and Recommendations in the Context of Exercise

**DOI:** 10.3390/jcm7060132

**Published:** 2018-05-31

**Authors:** Paul D. Loprinzi, Emily Frith

**Affiliations:** Exercise Psychology Laboratory, Physical Activity Epidemiology Laboratory, School of Applied Sciences, Department of Health, Exercise Science and Recreation Management, The University of Mississippi, MS 38677, USA; efrith@go.olemiss.edu

**Keywords:** consolidation, episodic, exercise, memory, physical activity, retrieval

## Abstract

There is evidence to suggest that biological sex plays a critical role in memory function, with sex differentially influencing memory type. In this review, we detail the current evidence evaluating sex-specific effects on various memory types. We also discuss potential mechanisms that explain these sex-specific effects, which include sex differences in neuroanatomy, neurochemical differences, biological differences, and cognitive and affect-related differences. Central to this review, we also highlight that, despite the established sex differences in memory, there is little work directly comparing whether males and females have a differential exercise-induced effect on memory function. As discussed herein, such a differential effect is plausible given the clear sex-specific effects on memory, exercise response, and molecular mediators of memory. We emphasize that future work should be carefully powered to detect sex differences. Future research should also examine these potential exercise-related sex-specific effects for various memory types and exercise intensities and modalities. This will help enhance our understanding of whether sex indeed moderates the effects of exercise and memory function, and as such, will improve our understanding of whether sex-specific, memory-enhancing interventions should be developed, implemented, and evaluated.

## 1. Overview

**Figure 1:**
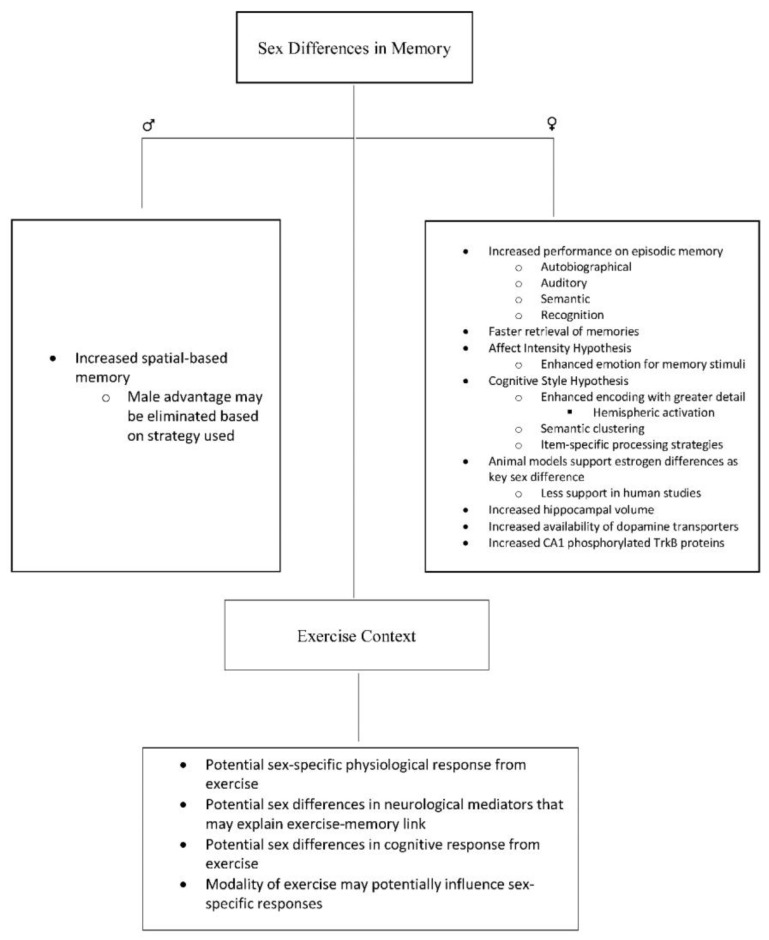
Parsimonious schematic of potential sex differences in memory and their implications for the exercise–memory link.

The purpose of this paper is to highlight the importance of evaluating sex-specific effects of exercise on memory function. In the narrative that follows, we will briefly discuss the unique role that sex has on memory function, indicate the dearth of studies evaluating whether sex moderates the effects of exercise on memory function, and discuss how molecular mediators (e.g., brain-derived neurotropic factor; BDNF) may have unique sex-specific effects on memory. Figure 1 displays a parsimonious schematic of the content of this review.

## 2. Sex Differences in Memory

In demented populations, females tend to have greater memory impairment than males [1], possibly as a result of APOE-ε4 allele presence (i.e., possession of at least one APOE-ε4 allele) [2], and faster whole brain and temporal lobe atrophic rates [3]. In non-demented populations, however, and in both cross-sectional and longitudinal designs, females tend to outperform males in episodic memory function [4,5,6], with a steeper age-associated decline among males (but not after age 60 [4,7], and with the degree of female superiority decreasing with advancing age [8]. Notably, however, this effect is not uniform across all types of episodic memory and cognitions [9]. Females tend to perform better than males in verbal-based episodic memory tasks, as opposed to spatial-based memory tasks [10]. Females generally access their memories faster than males [11], date them more precisely [12], and use more emotional terms when describing memories [13]. Superior verbal memory for females also appears to be independent of intelligence level [10]. Additionally, females also have greater specificity for events imagined to occur in the future [14].

In general, females outperform males on autobiographical memory (particularly with high retrieval support via verbal probing [13]), random word recall [6], story recall [15], auditory episodic memory [16], semantic memory (driven by superiority in fluency) [8], and face recognition tasks [10,17]. For race recognition tasks, females particularly have better recognition memory for female faces (and greater face perception [18,19]. This may be a result of females being more familiar with female faces [20], which aligns with other work showing that recognition memory is superior for individuals who are of the same ethnic background as themselves [21]. Females also have been shown to have greater scanning behavior at encoding [17], which may also contribute to their superior recognition memory.

Although it has been repeatedly reported that there is a male advantage for spatial-based memory tasks [6], it is becoming increasingly clear that spatial memory is not a unitary function [22]. For example, spatial rotation performance may be considered as a spatial working memory task [23]. Females tend to use an egocentric spatial strategy, which involves using local landmarks as directional cues. Males, however, tend to employ an allocentric strategy, which involves using a mental spatial map and orienting oneself in terms of absolute direction (north/south) [24]. The male advantage in spatial-based memory tasks can be eliminated or even reversed when salient landmark information is available throughout the memory task [25,26]. Further, studies have demonstrated greater female superiority in object location memory tasks [27]. Specifically, females appear to perform better than males at identifying target object exchanges, as opposed to displacements of objects to new spatial locations previously unoccupied by target images [28].

## 3. Potential Psychological Explanations for Sex Differences in Memory

Two commonly discussed hypotheses to explain potential sex differences in memory include the affect intensity hypothesis and the cognitive style hypothesis. The affect intensity hypothesis [29] states that females demonstrate superior memory function because the intensity of their responses to emotional experiences exceeds the average male reactions, which helps to facilitate the encoding of the memory trace. Further, emotional suppression is an emotional regulation strategy that may interfere with informational encoding or memory retrieval [30]. However, females appear to have greater episodic memory for neutral events/episodes [31], and some neuroimaging studies suggest that females do not process emotional memories more intensely than males (but there may be a lateralization effect) [32].

The cognitive style hypothesis suggests that females encode events in greater detail than males, whereas males encode the “gist” of events, as opposed to more specific details [33]. One speculation for this is the degree of hemispheric activation. For example, some work suggests that, for emotional memories, right hemispheric amygdala activity is associated with long-term memory retention for males, whereas activity of left hemispheric amygdalar activity is associated with long-term memories for females [32,34]. Some evidence indicates that the right hemisphere is biased toward processing the “gist” of memories, whereas the left hemisphere is biased toward finer detailed processing [35,36]. This, however, it not uniformly supported, as, although females recall longer, richer, and more evaluative narratives for autobiographical memories, males have been shown to recall more factual information [37]. Particularly with regard to autobiographic memories, parent–child reminiscence practices may partially explain the observed sex differences, as mothers tend to reminisce more elaborately and discuss emotional events in greater detail with their daughters when compared to their sons [38]. Further, and with regard to superior encoding of females [8], for a word list episodic memory task, males have been shown to have smaller across-trial gains and larger across-trial losses than females [39]. Thus, superior performance in females may be a result of gaining new items across trials rather than differences in retaining information. Such sex differences in verbal learning tasks may also be greater among lower performing individuals [39].

It is plausible that these sex differences may be due to differences in the organization of information. For example, females may be more likely to use semantic clustering in multi-trial verbal learning tasks [5,40], with semantic clustering shown to correlate with recall performance [41]. Thus, sex differences in verbal episodic memory appears to stem from differences in encoding rather than retrieval strategies [6,8,13]. Another potential explanation is that females may involve different item-specific processing strategies (e.g., semantic associates or rhymes of the words) [42]. Related to potential differences in the organization of information, language processing appears to be more bilateral in females, but more left-lateralized in males [43]. For example, when learning foreign words, females show bilateral activation of the fusiform cortex, while neural activity in this structure for males is left-lateralized [44]. Further support for male-specific left lateralization, is evidence indicating that following left temporal lobectomy, female’s verbal memory is spared, which is not the case for males [45,46].

## 4. Potential Biological Explanations for Sex Differences in Memory

Potential biological mechanisms explaining these sex differences include beneficial effects of estrogen on verbal episodic memory function (e.g., alterations in hippocampal neuronal activity; premenopausal women recruited left hippocampus more strongly than postmenopausal women [47,48]), differences (not supported by all studies [49]) in neuronal activity during memory retrieval of verbal information (e.g., males showing more activation in the left parahippocampal region, with females showing greater activation in the right dorsolateral prefrontal cortex [50,51], and differences in resting cerebral blood flow for temporal lobe regions (higher for females) [52]. With regard to the latter, greater relative cerebral blood flow in the left temporal pole for females has been associated with better episodic memory performance [52]. However, other work suggests that sex differences in episodic memory may not be entirely dependent on the hippocampus. For example, research shows that females perform better than males both before and after temporal lobe surgery, suggesting that sex differences in episodic memory are unrelated to group differences in hippocampal function [41]. This is supported by other temporal lobe surgical studies [53].

Although estrogen levels have been shown to correlate with greater episodic memory performance [54], estrogen exposure, however, has not been consistently shown to correlate with episodic memory performance in all human studies [55,56]. For example, in estradiol-matched females and males, females still performed better on episodic memory [57], suggesting that circulating estradiol is not the only contributing factor to sex differences in episodic memory. Importantly, though, matched circulating estradiol levels does not necessarily indicate similar estrogen receptor expression between males and females. Superior female performance on verbal episodic tasks has also been observed well before sexual maturity (e.g., in the preschool years) [58], further suggesting that sex differences are unlikely to be a result of sex hormones. However, in animal models, there is robust support for the effects of estrogen on memory-related parameters. For example, dendritic spine density in CA1 neurons varies across the estrous cycle [59,60,61]; ovariectomizing leads a 30% loss of spine density (which is reversed by estradiol therapy) [62]; menopause reduces the density of perforated synapse spines (correlating with recognition memory) [63]; and estrogen stimulates an increase in dopamine receptors [64], increases synaptic plasticity [65], and enhances neuronal growth [66].

Potential neuroanatomical explanations for the observed sex-differences in episodic memory includes females having larger volumes of hippocampal tissue (relative to brain size) [67], caudate nucleus [67], anterior cingulate gyrus [68], dorsolateral prefrontal cortex [69], and planum temporale [70]. However, males appear to have relatively larger volumes of the amygdala [71] and paracingulate gyrus [68]. Neurochemically, females appear to have a greater availability of dopamine transporters [72], and similarly, presynaptic dopamine levels in the striatum (involved in habit learning) appear to be higher in females than males [73]. Further, the neurotrophin, BDNF, plays a critical role in nearly all aspects of neuronal activity, including neuron survival, neurogenesis, branching of dendrites, release and production of neurotransmitters, synapse formation, and synaptic plasticity [74]. Female rats have been shown to have higher BDNF levels than male rats, in various nervous tissue, including the hippocampus, amygdala, and cortex [75,76]. In humans, hippocampal BDNF levels do not appear to differ among males and females, but females appear to have greater BDNF concentrations in the prefrontal cortex [77]. Studies demonstrate that enriched environments show greater neuronal BDNF expression in the hippocampus and prefrontal cortex in female mice [75,78]. In addition to both functional and sex-specific differences of BDNF, there is also evidence of sex-dependent signaling induced by BDNF. For example, female hippocampal CA1 regions have more phosphorylated TrkB proteins when compared to age-matched males [79]. Sex differences are also observable for NMDA expression [80] and long-term potentiation [81], key factors subserving memory function.

## 5. Sex Differences in Memory—Considerations for Exercise Research

Taken together, there is clear evidence that sex differences across various memory types are apparent. This may stem from sex differences related to neuroanatomical, neurochemical, neuroelectrical, and affect- and cognitive-specific processing strategies. Thus, at this point, it is unquestionable as to whether sex influences memory performance. This underscores the importance of evaluating sex-specific differences in memory function, and pertinent to this paper, evaluating whether behavior-induced differences in memory function vary across sex. Although there is emerging work demonstrating that both acute and chronic exercise may positively subserve memory function [82,83,84,85,86,87,88,89,90,91,92,93,94,95,96], particularly episodic memory, there is very little work in the exercise–memory domain evaluating potential sex differences [97].

Josefsson et al. (2012) conducted a prospective cohort study to investigate longitudinal correlates of verbal episodic memory changes, demonstrating that higher self-reported physical activity participation, female gender, advanced educational achievement, close social relationships, as well as being a carrier of the gene, catechol-O-methyltransferase (COMT), which is responsible for producing the dopamine-regulating enzyme, were more likely to maintain memory function across time, even when accounting for participant attrition [98]. The authors note that the specific memory test, the decision to utilize random thresholds when defining memory degradation, as well as investigation of linear changes across time, rather than both linear and nonlinear trajectories, limit the homogeneity of findings, thus preventing credible generalizations to broader populations. Moreover, cross-sectional data, while useful for identifying sex-specific relationships, do not allow for an investigation of meaningful causal linkages.

However, experimental work demonstrates that females may exhibit a preferential reliance on right parietal and prefrontal structures, which are expected to reflect a higher working-memory demand. This is assumed to corroborate the saliency of landmark recollections among women [99]. Specifically, females may track navigational patterns to a greater extent than men, recalling explicit, observable environmental clues to expedite successful navigation. Alternatively, men may recruit neural pathways within the left hippocampus during a spatial navigation assessment. As the hippocampus is intimately connected to optimal episodic memory function [100], researchers have posited that males may depend, almost exclusively, on episodic memory traces when performing spatial navigation tasks [101].

Episodic memory performance may also associate with heightened verbal memory evidenced among females [22]. For example, women have been shown to produce lengthier autobiographical accounts of previous experiences [102], which are often rife with precise details, such as accurate dates of event-specific occurrences [102]. Physical activity has also been suggested to support maintenance of endogenous estradiol and testosterone, which may reinforce episodic, verbal, and semantic memory among women [48]. Notably, a recent systematic review and meta-analysis described the role for potential sex-differences to influence episodic memory [103], demonstrating that aerobic and resistance training interventions did not appreciably differ by sex; although among studies recruiting primarily female participants, the positive effects of resistance training on episodic memory approached statistical significance. In contrast, sex-differences were evident for studies employing a combination of aerobic and resistance training activities, or “multimodal” physical exercise, providing rationale for a putative activation of multiple neuronal pathways to perhaps engender enhanced performance on episodic memory assessments.

To this end, it appears that males and females may integrate information from different neural networks, to arrive at similar behavioral targets, relative to overt learning and memory function.

This is of critical importance for several reasons, including the clear sex-specific effects on memory, unique sex-specific physiological responses to exercise [104], and importantly, memory-based neurological mediators (e.g., BDNF) may have a distinct effect on memory function based on the sex of the individual [105]. At this point, however, it appears that studies (among older human adults) who employ a greater proportion of females (vs. males) are more likely to observe exercise-related improvements in executive cognitive function, with uncertainty regarding memory function [103]. In rodents, however, when comparing male studies to female studies, the sex-dependent exercise-induced memory effect was influence by voluntary vs. forced exercise protocols [106]. These findings, coupled with very minimal work directly comparing male vs. female exercise-induced memory effects in a single study, underscores the importance of future carefully designed exercise–memory studies to evaluate sex-specific effects.

In conclusion, in this brief report, we highlight the potential sex differences in memory function, which extend to various memory domains, such as autobiographical memories, semantic memory, and memory recognition. Potential sex differences in memory are likely attributed to a multitude of factors, including various psychological (e.g., different processing strategies and learning strategies) and physiological parameters (e.g., brain structure, hormonal, and neurotransmitter differences). We wish to emphasize that future work evaluating the effects of exercise on memory function consider investigating whether sex moderates this effect. Such an effect is clearly plausible given the clear sex-specific effects on memory, exercise response, and molecular mediators of memory. Such work should be carefully powered to detect sex differences. Future research should also examine these potential exercise-related sex-specific effects for various memory types and exercise intensities and modalities. This will help enhance our understanding of whether sex indeed moderates the effects of exercise and memory function, and as such, will improve our understanding of whether sex-specific, memory-enhancing interventions should be developed, implemented, and evaluated.
